# Comparative Study of Collagen Gels Extracted from Different Sources

**DOI:** 10.3390/gels11110879

**Published:** 2025-11-01

**Authors:** Alina Elena Coman, Minodora Maria Marin, Ana Maria Rosca, Raluca Tutuianu, Madalina Georgiana Albu Kaya, Andreea Ionita, Rodica Roxana Constantinescu, Irina Titorencu

**Affiliations:** 1 National Research and Development Institute for Textiles and Leather, Division Leather and Footwear Research Institute, Collagen Department, 93 Ion Minulescu St., 031215 Bucharest, Romania; coman.alina27@yahoo.com; 2 Advanced Polymer Materials Group, University POLITEHNICA of Bucharest, 1-7 Polizu Street, 011061 Bucharest, Romania; 3 Institute of Cellular Biology and Pathology “Nicolae Simionescu”, 8 B. P. Hasdeu Street, District 5, 050568 Bucharest, Romania; anukya2003@gmail.com (A.M.R.); irina.titorencu@gmail.com (I.T.); 4 National Institute for Research & Development in Chemistry and Petrochemistry-ICECHIM, 202 Spl. Independentei, 060021 Bucharest, Romania; andreea.afilipoaei@yahoo.com; 5 National Research and Development Institute for Textiles and Leather, Division Leather and Footwear Research Institute, Biotechnologies and Environment Protection Research Department, 16 Lucretiu Patrascanu St., 030508 Bucharest, Romania; rodica.roxana@yahoo.com

**Keywords:** collagen gels, biomaterials, turkey collagen, American buffalo collagen

## Abstract

Collagen is well-known as an essential and structural protein in the body and is classified into many types, with different roles. Type I collagen is the most abundant, offering firmness, elasticity, and resistance to the skin. Starting from natural resources such as calf, American buffalo hide, turkey, and perch skin, this research aims to develop a comparative study between the porous matrices obtained from collagen, extracted in the form of gel, with potential medical use. The extracted collagen gels were analyzed for their proximate analysis. The structural conformation of the gels was confirmed using circular dichroism measurements. The extracted collagen gels were dried using a freeze dryer in the form of porous matrices, and structural analyses were performed using FT-IR. Further, the collagen scaffolds were assessed for biocompatibility using an XTT assay. The water swelling behavior, the morphology, and the thermal stability of the collagen matrices were determined. The collagen porous matrices presented good antimicrobial activity, especially COLL_P, which presented the highest inhibition zone, making them suitable for biomedical uses. Overall, this study provides a method for producing collagen matrices from various sources for biomedical applications.

## 1. Introduction

Collagen represents the primary structural protein in both human and animal bodies, accounting for approximately 30% of the total body protein weight, being present in the extracellular matrix and the connective tissues as the main fibrous protein constituent (skin, tendons ligaments, cartilage, bone, muscles, blood vessels, or cornea) and therefore performing various biological functions, providing tissue integrity, strength, and elasticity [1,2,3]. Until now, 29 distinct types of collagen have been identified. Type I is the most abundant and frequent type of collagen, widely used as a biomaterial in medicine, cosmetics, and frequently employed in the food industry, either in its native fibrillary form or upon denaturation, due to its multiple functional properties. These properties include biocompatibility; low antigenicity; biocompatibility; cell growth potential properties; and physical ability to be processed into a wide variety of products, including gels, porous scaffolds, fibers, and films [3,4,5].

To obtain extracts of type I fibrillar collagen, both fresh skin and industrial by-products from meat, fish, and poultry processing can be employed as raw materials. Raw materials, such as bovine, porcine, ovine, goat hide, chicken [2], fish skin [6], turkey tendon [3], and others, are abundant and suitable for extracting this valuable protein using methods such as acid extraction, alkali extraction, or enzymatic hydrolysis [7]. The molecular structure of type I collagen is defined by three polypeptide α-chains, which form a unique conformational structure of a triple helix. Hydroxyproline is found in all collagen types and, along with proline and glycine amino acids, contributes significantly to collagen triple-helix stability through interchain hydrogen bonds [1,8].

Depending on the field of use, the triple-helical structure of collagen is very important for its biological activity, including cell adhesion, as well as for good mechanical properties [9]. The triple-helical structure of type I native collagen can be affected by various factors (temperature, pressure, pH, chemical concentration (acid or alkali), or enzymes), leading to denaturation [10]. Collagen extracts may be denatured or undenatured, based on their structure. Collagen extracts, in the form of gels, pastes, or solutions, that maintain the triple-helical structure during extraction can be involved in biomaterials synthesis. Biomaterials based on type I fibrillar collagen, including medical devices, artificial implants, drug delivery systems, and scaffolds for tissue regeneration, are intensively used nowadays [8,11]. When collagen undergoes partial denaturation, under the influence of physical or chemical factors, its native triple-helical conformation breaks down to the level of the secondary structure, leading to the formation of gelatine (short polypeptide chains), whereas with further denaturation, until the primary structure level, collagen is degraded into hydrolysates (amino acids) [8,10].

Gelatine is used in the food sector as a thickener, stabilizer, emulsifier, gelling agent, and moisture absorber. It improves texture and fastens flavors in sauces, desserts, and low-fat dairy substitutes [12,13]. With respect to biomedical sectors, gelatine is used for tissue engineering and medical devices [14,15,16], as a plasma expander in cases of blood loss [17], as a protective shell for pills and capsules [18], and as the main ingredient in hemostatic sponges and wound dressings [6]. Collagen hydrolysates are employed in the development of pharmaceutical [19], cosmetic [20], and nutraceutical products [12].

In the literature, there is abundant information regarding the extraction of collagen with potential uses as biomaterials from different skin sources; however, as far as we know, there are no publications regarding collagen extraction, in the form of gels, from American buffalo and turkey skin. Moreover, there are only a few articles regarding comparative studies for collagen extracted from different animal skin sources [21,22]. Therefore, in this study, we developed collagen porous matrices with potential biomedical applications, extracted from different natural sources, and performed a comparative study between them. Type I collagen was extracted from natural resources, including calf, American buffalo hide, turkey, and perch skin. For all skin types, the extraction was carried out in an acidic medium, yielding collagen in gel and gelatin form. The method for collagen extraction from calf hide was set up according to our technology previously described [4]. A similar technique was employed for collagen extraction from American buffalo hide and turkey skin, whereas the procedure for extracting collagen from perch skin has been described in detail in our previous study [6].

The extracted gels were subjected to physical–chemical and structural analyses. To develop collagen porous matrices, the gels derived from calf, buffalo hide, turkey, and perch skin were freeze-dried. Since the ability of these porous matrices to control moisture is crucial, their swelling behavior, assessed via water absorption analysis, and morphology, assessed via SEM analysis, were investigated. Furthermore, the collagen porous matrices underwent microbiological assessment, structural analysis (CD and FT-IR), and biocompatibility evaluation. The results highlight a novel approach to producing native collagen from various sources, which can be used as raw materials for biomedical applications.

## 2. Results and Discussion

### 2.1. Characterization of Collagen Gels Extracted from Different Sources

The purity, molecular and morphological structure, and ability to be processed into the required shape determine the characteristics of collagen gels used as biomaterials for biomedical applications.

Collagen, in the form of gels, was extracted through acidic treatment from different sources of skin, such as calf (COLL_C), American buffalo (COLL_B), turkey (COLL_T), and perch (COLL_P). Collagen extracted from fresh hide or skin of different animals, in an acidic environment, was physically–chemically and structurally analyzed.

#### 2.1.1. Physical–Chemical Analysis

The collagen gels were characterized using physical-chemical methods such as dry substance, ash, total nitrogen, protein content (calculated from nitrogen content), and pH. The results are presented in Table 1.

All the collagen gels present a relatively high concentration of collagen, in the range of 1.15–1.90%. Usually, in order to obtain collagen biomaterials, gels with a concentration of 0.5–2% are used [4,23,24]. As presented in Table 1, the highest dry matter content was achieved using gel COLL_C, derived from calf hide. The dry matter content reflects the amount of collagen present in the sample, standardized to 100 g, with the remainder comprising water.

All samples exhibited elevated dry matter and protein content, indicating favorable compositional characteristics for gels. The total nitrogen content represents the amount of all compounds containing nitrogen present in gel, including proteins, amino acids, and organic and inorganic nitrogen-based compounds [25,26]. Collagen extracted from perch skin shows a lower total nitrogen content, while the other samples show a similar percentage of total nitrogen.

Collagen extraction is primarily influenced by the specific treatment applied to animal hides/skins, which aims to eliminate non-collagenous substances such as minerals, lipids, and other impurities. The removal of these components occurs throughout the extraction process, beginning with the pretreatment of raw materials and continuing through extraction and purification steps [2]. A high degree of sample purity is evidenced by the ash and fat content [20], which was undetectable for all the gels; hence, all the samples exhibit a high degree of purity.

Collagen samples were found to be acidic, which is typical for acid-extracted collagen. Acidic pH values are advantageous because they prevent collagen degradation and increase the stability of the final product [27]. All gels exhibit a pH between 2 and 4.2 pH units.

Among all the samples, GEL_COLL_C displayed the highest levels of protein and dry matter, followed by COLL_B and COLL_T. The lowest dry matter and protein content was recorded in the COLL_P sample. These findings were further corroborated by circular dichroism analysis.

#### 2.1.2. Circular Dichroism Spectroscopy (CD)

The CD spectra of collagen samples are highly dependent on their conformation, making CD a useful technique for monitoring collagen conformational changes, induced by environmental factors or irreversible denaturation [28]. According to the literature, the CD spectra of type I native collagen show a positive band at 220 nm, which decreases during denaturation, and a negative band at 198 nm, characteristic of a triple-helical conformation, and a crossover point at 214 nm [9,29,30,31,32]. During complete thermal denaturation, the positive peak disappears, and the negative peak intensity decreases [9,29].

**Figure 1:**
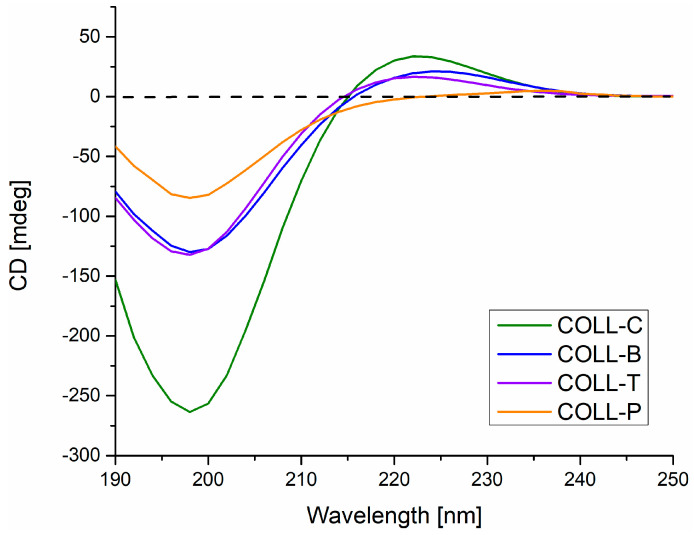
Circular dichroism spectra for type I collagen, extracted from different skin sources.

Figure 1 presents the CD spectra of type I collagen samples extracted from different skin sources. Spectra of collagen isolated from calf hide, COLL_C, present a weak positive maximum peak at 222 nm and a negative minimum absorption band at 198 nm, with a crossover point at 214.8 nm, characteristic of the typical triple-helical conformation of collagen in the form of gel. Very similar results were obtained for COLL_B and COLL_T samples, with a positive band at 223 and 222 nm, respectively, a negative band at 198 nm, and a crossover point of 215.5 nm for COLL_B and 214.5 nm for COLL_T, respectively.

Therefore, CD spectra for COLL_C, COLL_B, and COLL_T suggest a preserved tertiary structure of non-denatured collagen, confirming the presence of a native triple-helix conformation [1,33] maintained during the extraction process.

Regarding COLL_P, as expected from our previous study [6], the COLL_P sample shows a positive peak at 236 nm and a negative band at 198 nm, with an intensity reduced to about half that of the other samples [16,34]. The crossover point was red-shifted to 222 nm, denoting partially denatured collagen with random coil configuration, suggesting a gelatine structure, in agreement with other studies [35].

**Figure 2:**
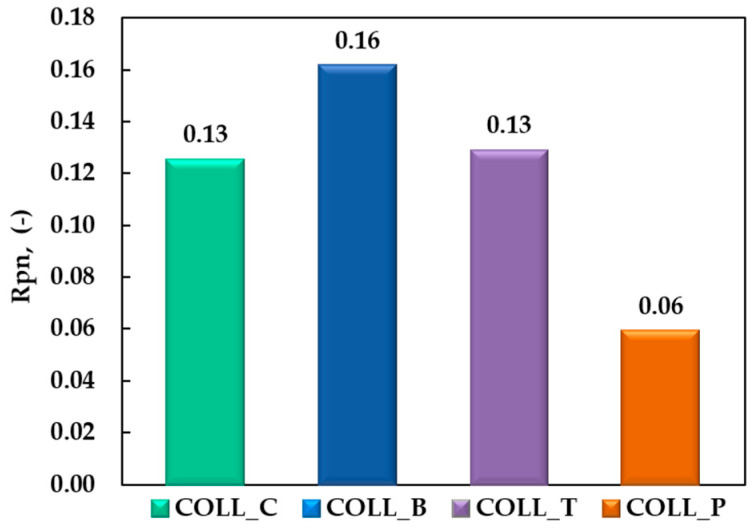
Graphical representations of Rpn for collagen gels extracted from different sources.

Another important aspect revealed from the CD spectra of extracted collagen gels is shown in Figure 2, which presents the Rpn parameter (the ratio of the absolute values of the positive and negative peak intensities), which is essential for determining the degree of collagen packing as triple-helical molecules.

According to other researchers, the Rpn value around 0.12 suggests that type I collagen molecules maintained their native triple-helix structure [36,37].

For samples COLL_C, COLL_B, and COLL_T, the Rpn values confirm the triple-helix structure of collagen extracted from calf and American buffalo hides and turkey skin, which was kept during the extraction process. The Rpn values for COLL_C and COLL_T were 0.13, and those for COLL_B were 0.16, reflecting a superior degree of collagen packing as a triple helix in gels, compared with native collagen, where Rpn is 0.12.

Partial collagen denaturation leads to conformational modification in the molecular structure of collagen, with decreasing intensity and the position of positive and negative peaks in CD spectra of the sample, by shifting the crossover point toward red and decreasing Rpn values, whereas complete denaturation completely eliminated the positive peak and decreased the intensity of the negative peak, simultaneously with its shift toward red in the UV spectrum [29,31,38].

As illustrated for the COLL_P sample in Figure 2, the Rpn value is 0.06, below 0.12, indicating partial denaturation of the collagen extracted from perch skin and the gelatine structure, as expected, from our previous study [6]. Actually, the reason for the decreased Rpn value for COLL_P is consistent with partial loss of triple-helical structure and can be attributed to the intrinsically lower thermal stability (shrinkage/denaturation temperature) of fish-derived collagen, which is linked to its lower imino acid (proline + hydroxyproline) content compared with mammalian collagen [39].

Consequently, even mild processing techniques or room-temperature conditions can lead to partial denaturation, reflected in the low Rpn value. These results align with literature reports describing reduced helical stability and lower denaturation temperatures in fish collagen compared to bovine or porcine sources [40,41,42].

The circular dichroism spectroscopy results are consistent with the FT-IR spectra of the collagen porous matrices presented later.

### 2.2. Characterization of Collagen Porous Matrices

The pH and the concentration of the gels extracted from various sources (COLL_C, COLL_B, COLL_T, and COLL_P) were adjusted with an alkaline solution (NaOH 0.1 M) and distilled water under mechanical stirring. The concentration of gels was decreased to 1% (*w*/*v*) for all the samples, and the pH was increased to a physiological pH to promote cell growth. Then, the gel samples were lyophilized according to a process previously described [43] to remove the water and to produce the corresponding porous matrices (collagen sponges), which were further analyzed in order to determine their potential uses as biomaterials.

#### 2.2.1. Structural Analysis of Collagen Porous Matrices—FT-IR Spectra

FT-IR spectroscopy of collagen porous matrices, extracted from different sources, highlights the secondary and tertiary structure of collagen and identifies the functional groups.

**Figure 3:**
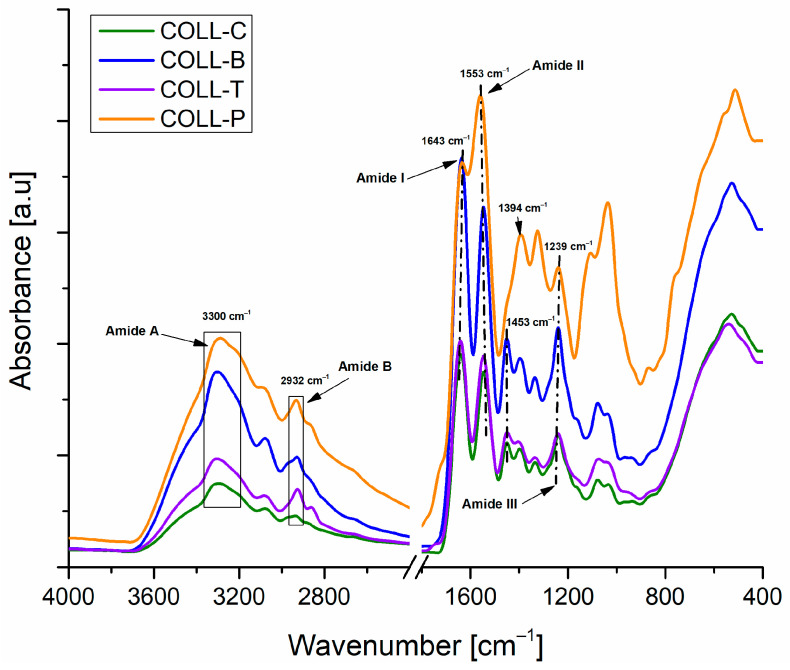
FTIR spectra of collagen gels extracted from different sources.

All COLL matrices’ FT-IR spectra, as can be noticed in Figure 3, were similar to those of collagen from sea sources [27,44], turkey feet [45], and camel hoof [46]. All FT-IR spectra exhibit five main absorption bands, in agreement with the literature, specific to the collagen structure: amide A band at about 3300 cm^−1^; amide B, with peaks at 3080–2900 cm^−1^; and amide I, II, and III at about 1650, 1554, and 1240 cm^−1^, respectively [47,48,49].

The amide A band, present at 3300 cm^−1^ for all COLL sponges, is related to the stretching vibrations of N–H groups, coupled with hydrogen bonds from the carbonyl group in the peptide chain [1,20]. Amide B signals were found at wavenumbers of 2932 cm^−1^ for all the samples and represent the symmetrical or asymmetrical stretching vibration of the CH_2_ group [1,27]. The amide I peak, observed at 1643 cm^−1^ for all collagen sponges and characteristic of the secondary structure of proteins, is associated with the stretching vibrations of carbonyl bonds along the polypeptide structure. The amide II peak, for all collagen matrices, was observed at 1553 cm^−1^, resulting from N–H bending vibration, coupled with the stretching vibration of C–N.

The signal for amide III at 1239 cm^−1^, related to C–N vibrational stretching and N–H bending, is associated with the triple-helical structure of collagen [1,6,20,50].

With the aim of evaluating the integrity of triple-helix structure, for all collagen porous matrices, the FT-IR absorption ratio of Amide III to 1450 cm^−1^ band (A_III_/A_1450_) was determined.

The band around 1450 cm^−1^ corresponds to the C-H stretching and bending vibrations from CH_2_ groups present in the pyrrolidine ring of proline and hydroxyproline due to the molecular structure of collagen samples [51,52]. For COLL_C, COLL_B, and COLL_T, the band was present at 1453 cm^−1^, whereas for COLL_P matrices, the band was displaced at 1394 cm^−1^. Hence, as in the case of native type I collagen, COLL_C, COLL_B, and COLL_T samples proved to have an amide ratio close to unity (A_III_/A_1450_ = 1.08, 1.05, respectively 1), while a lowered amide ratio (A_III_/A_1450_ = 0.90) was found in the case of the COLL_P sample, indicating partial denaturation of the collagen structure.

The results obtained from FT-IR analysis, with respect to the triple-helix structure of the extracted gels, are correlated with circular dichroism spectroscopy analysis.

Similar bands in the amide regions to those found in this work for all collagen samples were reported for collagen hydrolysates from buffalo skin [53] and for turkey tendon collagen [3].

#### 2.2.2. Swelling Behavior of Collagen Porous Matrices

**Figure 4:**
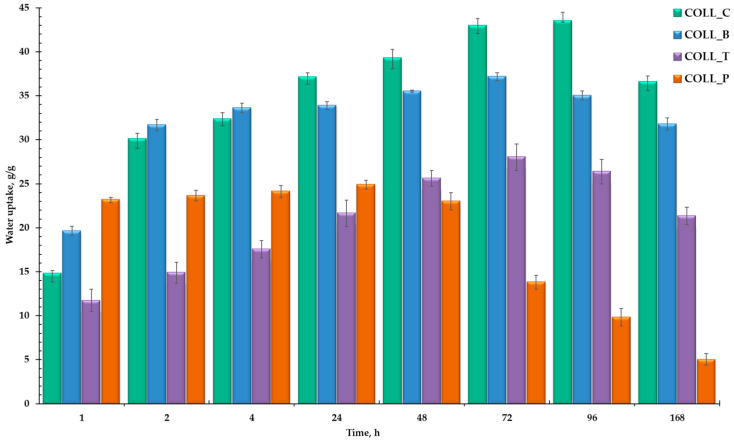
Water uptake ability of collagen matrices immersed in distilled water at room temperature for 168 h.

The swelling behavior, or water uptake properties, of collagen biomaterials is a crucial factor for their potential medical applications, such as wound healing or tissue engineering. The ability of collagen spongy matrices to absorb water is essential for proper fluid retention, facilitating drug release and influencing the mechanical properties of collagen scaffolds [6,50,54]. Figure 4 shows the water uptake of collagen sponges, extracted from different sources, when hydrated for seven days. The pore structure of collagen porous matrices directly influences the collagen sponge’s water absorption.

The samples showed different swelling abilities, using a consistent amount of water (~17–43 g/g). All the collagen sponges absorbed a significant amount of water shortly after immersion and very fast in the first hours, with COLL_P reaching equilibrium after 24 h with 25 g/g of water absorbed. After 48 h, the weight of the COLL_P sample started to decrease, proving that the degradation process began. This result is in accordance with the literature [55], demonstrating that gelatine extracted from fish leads to materials with a lower swelling degree than collagen extracted from mammals [14].

As expected, COLL_C maintained its integrity even after 96 h, absorbing the highest amount of water, 43 g/g of water. After four days, the sample started to dissolve slowly. COLL_B followed the same pattern, with water uptake increasing constantly for 72 h, reaching equilibrium at 37 g/g of water absorbed. COLL_T seems to be denser than COLL_C and COLL_B and absorbed a smaller amount of water, reaching 28 g/g of water after three days. Starting on the fourth day, after 96 h, COLL_C, COLL_B, and COLL_T sponges started to degrade, showing a decrease in water uptake.

To conclude, the swelling capacity of collagen porous matrices was high for all samples, showing a different tendency, depending on the specific biomedical application, wound healing or tissue engineering. The porous structure of the samples will be further confirmed by SEM analysis.

#### 2.2.3. Morphological Analysis of Collagen Porous Matrices

**Figure 5:**
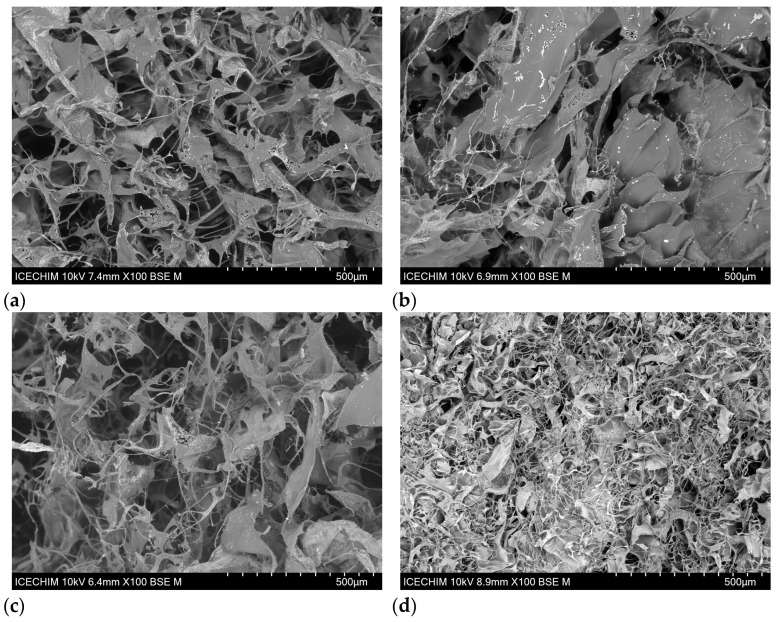
SEM images of collagen porous matrices extracted from different animal sources (magnification x100): (**a**) COLL_C, (**b**) COLL_B, (**c**) COLL_T, and (**d**) COLL_P.

SEM analysis was performed to investigate the morphology of the freeze-dried collagen matrices extracted from different animal sources, and the micrographs are presented in Figure 5.

For collagen biomaterials, the pore structure, observed by SEM analysis, directly influences the sponge’s water absorption capability. A porous, interconnected network is crucial for collagen biomaterials, especially for tissue engineering scaffolds. Porous matrices promote cell infiltration, migration, and proliferation and facilitate the transport of nutrients and oxygen necessary for tissue growth and vascularization. The pore’s size, morphology, and interconnectivity are key factors for mimicking the natural tissue environment, enhancing the biological response, and promoting the functional recovery or regeneration of the tissue [50,56,57].

According to Figure 5, all the collagen sponges exhibited a porous structure, with pore sizes between 40 and 143 mm, optimal for collagen biomaterials [50,57,58], in concordance with the water absorption results presented above. COLL_P, Figure 5d presents a denser structure, with small pores (40–73 mm), interconnected, in accordance with water absorption results. COLL_C, COLL_B, and COLL_T present similar porous structures, with large pores of comparable dimensions. COLL_C presents pore sizes ranging from 60 to 143 mm, while COLL_B and COLL_T exhibit pores with sizes between 60 and 116 mm.

SEM images show, for all the samples, the presence of the alkaline solution (NaOH 0.1 M) used to adjust the pH and the concentration of the gels extracted from various sources. The presence of sodium is uniformly distributed in the sample’s surface, demonstrating the homogeneity of the collagen samples.

#### 2.2.4. Collagen Sponge Thermal Analysis

**Figure 6:**
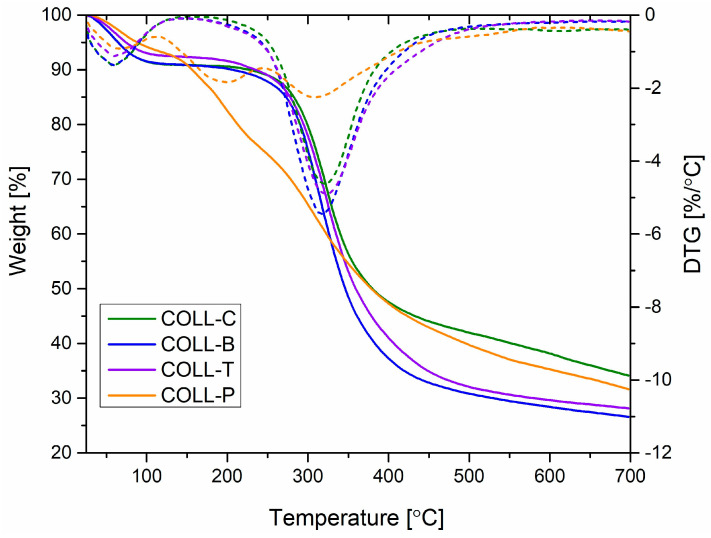
TG and differential TGA (DTG) curves for collagen sponge samples.

To evaluate the thermal stability of the collagen sponges from different sources, under optimal conditions, thermogravimetric analysis (TGA) was employed, and the results are presented in Figure 6. All collagen matrices showed an initial weight loss from 25 °C up to 100 °C (stage 1), with a maximum degradation rate around 55–60 °C, corresponding to the presence of moisture and evaporation of water molecules from inside the collagen fibers [59,60]. As can be seen in Table 2, in this stage, the mass loss is between 5.80 and 8.50% for all the samples. The second (100–300 °C) and third stage (300–500 °C) represent thermo-oxidative processes and are attributed to collagen molecule decomposition, the breakage of protein chains associated with the triple-helical structure, and peptide bond cleavage. After 500 °C, combustion of the residual collagen organic matrix takes place [5,59,60]. As can be seen in Figure 6, COLL_P, extracted from perch skin, presents the highest weight loss of ~29% in the second stage of degradation, starting to decompose earlier than the other samples. It is followed by COLL_B from American buffalo hide, COLL_T from turkey skin, and the lowest mass loss in the second stage is achieved by COLL_C from calf hide, as expected.

Therefore, the thermal transition and weight loss behavior shown by collagen sponges from perch skin, the COLL_P sample, was fast and sharp from 100 to 500 °C, whereas the collagen sponges from calf, buffalo, and turkey skin showed a slow, gradual weight loss for a similar region, but with the shift of the thermal step to a higher temperature. For example, the end point, or temperature of the maximum decomposition rate, T_max_ (DTG) from Table 2, of 309 °C for COLL_P shifts to 323 °C for COLL_T, 322 °C for COLL_C, and 315 °C for COLL_B, indicating greater thermal stability for the latter samples, in accordance with CD, FT-IR, and water uptake analysis of collagen porous matrices from different sources. Due to the gelatinous structure of the COLL_P sample, the denaturation process began earlier compared with the other samples. These results are in line with previous thermogravimetric analysis studies conducted on fish gelatine [12,15].

The residue at 700 °C was ~26–34%, with lower values for COLL_B and COLL_T.

#### 2.2.5. Collagen Porous Matrices Microbiological Analysis

The antibacterial activity of biomaterials based on collagen is very important. For example, wound dressing materials should be impermeable to external bacteria; if bacteria penetrate the wound, it may lead to severe infection and delay healing [61,62]. In collagen-based composites for tissue engineering, infection represents a major risk; therefore, these materials should exhibit antibacterial activity.

**Figure 7:**
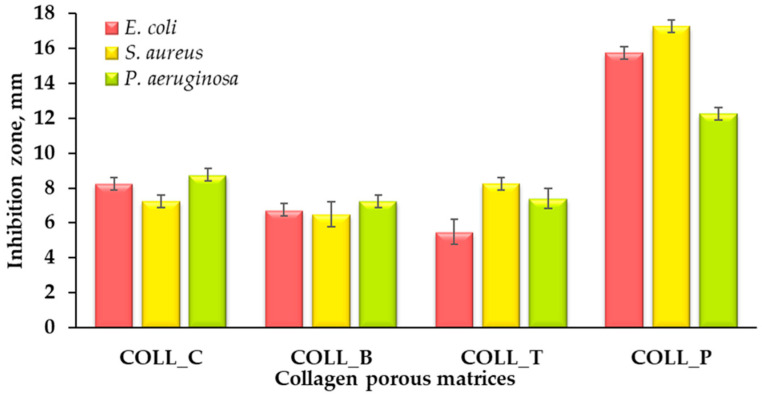
Antibacterial activity of collagen sponges against *E. coli*, *S. aureus*, and *P. aeruginosa*, measured using disc diffusion method.

Figure 7 represents the antibacterial activity of collagen porous matrices against the primary pathogens associated with infections: Gram-negative bacteria—*Escherichia coli* (*E. coli*) and *Pseudomonas aeruginosa* (*P. aeruginosa*), and Gram-positive bacteria—*Staphylococcus aureus* (*S. aureus*).

The collagen sponges possess antimicrobial activity, proving the efficiency of the collagen porous matrices for biomedical use. The highest inhibition zone for the pathogens tested was achieved by COLL_P, followed by COLL_C, COLL_B, and COLL_T, which present similar antibacterial activity. It is well known that marine collagens represent a promising source of antimicrobial material [19,63].

Regarding microbial contamination, in accordance with the current edition of the European Pharmacopoeia (Ph. Eur.), the 11th Edition, the scope of the determination is to identify the microbial contamination of the collagen sponges. Collagen sponges are considered non-sterile dosage forms. The microbial contamination control aims to determine the total number of aerobic microorganisms (TAMCs) or the absence of pathogenic microorganisms (TYMCs).

The results presented in Table 3 were reported as total aerobic microbial count (TAMC), representing the average CFUs determined on the Soybean–Casein Digest Agar medium, and the total combined yeasts and molds count (TYMC), representing the average CFUs determined on the Sabouraud Dextrose Agar medium.

In the harmonized text of pharmacopoeia monographs concerning the microbiological quality of non-sterile dosage forms, for pharmaceutical use, the allowance limits for TAMC are ≤1000 CFU/g and for TYMC are ≤100 CFU/g [64,65,66].

The microbial contamination of collagen sponges from different sources, presented in Table 3, shows that TAMC and TYMC values do not exceed the limits of admissibility provided by the current edition of European Pharmacopoeia. COLL_P counts the smallest colonies of fungi and bacteria, followed by COLL_C, COLL_B, and COLL_T, in accordance with the antibacterial activity. Moreover, the collagen samples do not enable the development of aerobic germs for any of the tested bacteria, *E. coli*, *S. aureus*, and *P. aeruginosa*.

The values found for TAMC and TYMC (under 100 CFU/g), and the absence of *E. coli*, *S. aureus*, and *P. aeruginosa,* permit us to affirm that the collagen porous matrices, obtained from different animal sources, are suitable for biomaterials with medical use.

#### 2.2.6. Evaluation of the Biocompatibility of Various Collagen Gels with MG63 Cells

**Figure 8:**
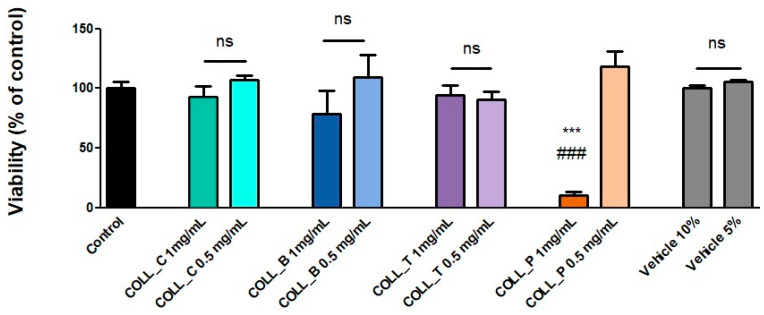
Cell viability for MG63 cells with 72 h of incubation in the presence of collagen gels (*** *p* < 0.001 versus control, ### *p* < 0.001 versus COLL_P 0.5 mg/mL, ns—nonsignificant). The differences between all the other samples, except for COLL_P 1 mg/mL, were not statistically significant versus control or vehicles (water).

All the tested collagen gel solutions were determined to have good compatibility with MG63 cells, with the notable exception of the higher concentration (1 mg/mL) of COLL_P. However, when further diluted to 0.5 mg/mL, this sample was also shown to support good cell viability, as determined by the XTT assay (Figure 8). The vehicle, which was water, was also tested to see if it could affect the viability of MG63 cells when used in the quantities necessary for making the sample solutions. As can be seen in Figure 8, it did not impact viability. For COLL_C, COLL_B, and COLL_T, there was no statistically significant difference in comparison to the control cells grown in culture medium. Moreover, both tested concentrations show similar support for cell viability, since no statistical significance was obtained for the small variations between each condition.

## 3. Conclusions

This research focuses on collagen extracted from different sources, such as calf, American buffalo hide, turkey, and perch skin, in the form of gel/gelatine, in order to obtain biomaterials based on type I collagen.

The extracted collagen gel samples were analyzed via proximate analysis, which showed a relatively high concentration of collagen, between 1.15 and 1.90%. Ash and fat content were undetectable for all collagen samples, demonstrating a high degree of purity and efficient removal of the chemicals used during the extraction process.

The results obtained from FT-IR analysis, regarding the triple-helix structure of the extracted gels, were correlated with circular dichroism spectroscopy analysis, showing that samples COLL_C, COLL_B, and COLL_T maintained the triple-helix conformation during the extraction process, while the COLL_P sample presents a random coil configuration, suggesting a gelatine structure.

The swelling capacity of collagen porous matrices was high for all samples, showing a different tendency, depending on the specific biomedical application: wound healing or tissue engineering. The porous structure of the samples was confirmed using SEM analysis.

With respect to the thermal stability of collagen porous matrices, the thermal transition and weight loss behavior presented by collagen sponges extracted from perch skin, COLL_P sample, was fast and sharp from 100 to 500 °C, whereas the collagen sponges from calf, buffalo, and turkey skin showed a slow, gradual weight loss for the similar region, but with a shift of the thermal step to a higher temperature.

The microbiological analysis showed that all collagen sponges possess antimicrobial activity, proving their efficiency for biomedical use. The highest inhibition zone for the pathogens tested was achieved by COLL_P, followed by COLL_C, COLL_B, and COLL_T, which present similar antibacterial activity. Regarding the microbial contamination of all collagen sponges from different sources, TAMC and TYMC values do not exceed the limits of admissibility provided by the European Pharmacopoeia

All the collagen gel solutions tested showed good compatibility with MG63 cells, a human osteosarcoma cell line, CRL-1427, ATCC, with the notable exception of the higher concentration (1 mg/mL) of COLL_P, as well as the microbiological testing results, proving the efficiency of collagen extracted from marine resources.

The results obtained reveal an innovative path to produce native collagen extracted from different sources. All the extracted collagen gels exhibit a set of desirable properties for biomedical applications. Their antimicrobial activity, good biocompatibility with MG63 osteoblast-like cells, and well-defined porous structure, revealed by SEM and water uptake analyses, highlight their potential as multifunctional biomaterials.

As a result of these characteristics, collagen sponges are particularly suitable for bone tissue engineering, osteogenic cell growth and tissue integration support, and wound healing applications, where the prevention of infections and tissue regeneration are essential.

## 4. Materials and Methods

### 4.1. Materials

Type I collagen (COLL) was extracted from natural resources, including calf (C) and American buffalo (B) hide and turkey (T) and perch (P) skin. For all skin types, the extraction was carried out in an acidic medium, yielding collagen in gel form. The method for collagen extraction from calf hide was set up according to our technology previously described [4]. The same technique was employed for collagen extraction from American buffalo hide and turkey skins, whereas the procedure for extracting collagen from perch skin was described in detail in our previous study [6]. The acids used for collagen extraction were L (+) ascorbic acid purchased from Scharlau (Sentmenat, Spain) and HCl 37% from Remed Prodimpex (Bucharest, Romania). Sodium hydroxide, analytical grade, was supplied by Chemical Company (Iasi, Romania). Distilled water served as the solvent for the experimental preparation of samples.

### 4.2. Methods

#### 4.2.1. Extraction of Collagen Gels from Different Sources

**Figure 9:**
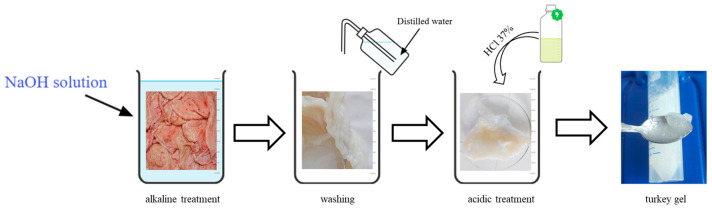
Schematic representation of collagen extraction process, in form of gel, from turkey skin.

Briefly, the method of extraction from calf hide is characterized by a short time treatment in a strong alkaline medium in the presence of neutral salts, followed by the complete dissolution of the hide in an acidic solution (HCl was used). The extraction method employed for collagen from American buffalo and turkey skins resembles that of calf hide, but the technology is patentable and cannot be described in detail. Figure 9 shows the scheme of collagen extraction starting with turkey skin.

#### 4.2.2. Proximate Analysis of Extracted Gels

The proximate analysis, including the dry substance, ash, and protein content of the extracted gels, was determined according to Romanian standard methods SR EN ISO 4684:2006 [67], SR EN ISO 4047:2002 [68], and SR ISO 5397:1996, respectively [69]. Total nitrogen content was determined using the Kjeldahl method. The pH of the gels was determined potentiometrically according to SR EN ISO 4045:2018 [70] using a laboratory pH meter InoLab pH Level 1 (InoLab, Oostkamp, Belgium).

#### 4.2.3. Structural Analysis of Extracted Gels

The preservation of the triple-helical structure in the collagen samples was assessed using circular dichroism (CD) spectroscopy. Spectral data were collected with a Jasco Model J-1500 spectrophotometer (JASCO, Tokyo, Japan), employing a quartz cylindrical cuvette with a 10 mm path length, containing 2 mL of aqueous collagen solution. CD spectra were recorded with scanning wavelengths from 190 to 260 nm at a scan rate of 100 nm/min.

#### 4.2.4. Preparation of Collagen Sponges

**Figure 10:**
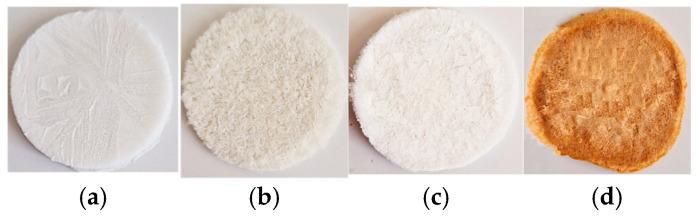
Collagen porous matrices and extracted gels after lyophilization process: (**a**) COLL_C, (**b**) COLL_B, (**c**) COLL_T, and (**d**) COLL_P.

The pH and the concentration of the extracted collagen gels were adjusted with a solution of NaOH 0.1 M and distilled water under mechanical stirring. The gel’s concentration was decreased to 1% (*w*/*v*) for all samples, and the pH was increased to a physiological pH (7.2–7.4) to promote cell growth [6]), poured into a Petri dish, and freeze-dried for 48 h [43]. The result was the collagen sponges (porous matrices, presented in Figure 10). These adjustments were made to enable a reliable comparison between the collagen samples by standardizing the concentration and the pH.

#### 4.2.5. Structural Analysis of Collagen Porous Matrices

To investigate the chemical bonding structure within the collagen sponges, the ATR-FTIR technique was employed. Fourier-Transform Infrared (FTIR) spectroscopy (Bruker, Billerica, MA, USA) was conducted using a Bruker Vertex 70 spectrometer (Billerica, MA, USA) equipped with an attenuated total reflectance (ATR) accessory. For each prepared formulation, FTIR spectra were acquired in the ATR mode, at a resolution of 4 cm^−1^, over the spectral range of 400–4000 cm^−1^.

#### 4.2.6. Swelling Behavior of Collagen Porous Matrices

The swelling behavior, namely, water uptake capacity, of collagen porous matrices was determined using the weight method [6], weighing a piece of dry porous matrix, of about 1 cm^3^, before immersion in distilled water (*W_d_*). After weighing the dry sponges, the samples were subsequently placed in 2 mL of distilled water at room temperature for seven days. At regular periods of time, the swollen matrices were weighed (*W_s_*), and the capacity to retain water was calculated using Equation (1). The experiment was performed in triplicate. For every sample, the mean and standard deviation were calculated.

(1) $$Swelling\;behaviour\;\left(g/g\right)=\frac{W_{s}{-W}_{d}}{W_{d}}$$

#### 4.2.7. Morphological Analysis of Collagen Porous Matrices

The morphological characteristics of the collagen porous matrices were investigated using a Hitachi TM4000Plus microscope (Hitachi, Tokyo, Japan) in the backscattered electron (BSE) mode at an accelerating voltage of 10 kV. The collagen samples were analyzed in the form of uncoated sponges.

#### 4.2.8. Collagen Sponges Thermal Analysis

Thermogravimetric analysis (TGA) of the collagen samples was performed in triplicate using a NETZSCH TG 209 F1 Libra instrument (NETZSCH-Gerätebau GmbH, Selb, Germany) under a controlled nitrogen atmosphere at a flow rate of approximately 20 mL/min. The samples were heated from 25 to 700 °C at a constant rate of 10 °C/min. Each measurement was carried out on sample masses ranging between 5 and 6 mg.

#### 4.2.9. Collagen Porous Matrices Microbiological Analysis

The antimicrobial assay was performed using standard strains from the INCDTP, Microbiology Department collection, as follows: *Escherichia coli* (ATCC 11229), *Staphylococcus aureus* (ATCC 6538), and *Pseudomonas aeruginosa* (ATCC 27853).

The qualitative screening of the antimicrobial properties was performed using an adapted spot diffusion method [71]. Bacterial suspensions at 1.5 × 10^8^ CFU/mL (corresponding to a 0.5 McFarland standard density) were obtained from 24 h microbial cultures, developed on Muller-Hinton agar (MHA). The plates were left at room temperature to ensure the equal diffusion of the compound in the medium, and were then incubated at 37 °C for 24 h. The presence of clear zones around the collagen sponges was analyzed for the existence of antibacterial activity after incubation of the plates for 24 h at 37 °C. The diameter of the inhibition zone was measured in mm and calculated using the following equation:

(2) $$H\;\left(mm\right)=\frac{D-d}{2}$$

where *H* represents the inhibition zones, mm in diameter; *D* is the total diameter of the disc and the area of inhibition, in mm; and *d* represents the diameter of the disc, in mm.

The antimicrobial assay was performed in duplicate. For every sample, the mean and the standard deviation were calculated.

For the determination of microbial contamination, the total aerobic microbial count (TAMC) and total yeast and mold count (TYMC) assays were performed. Casein Soya Bean Digest Agar (Novachim, Bucharest, Romania) was employed for total aerobic microbial count (TAMC), and Sabouraud Dextrose Agar (Novachim, Bucharest, Romania) was used for total fungal count. The plates were incubated at 30–35 °C for 1–2 days for TAMC and at 20–25 °C for 3–5 days for TYMC.

#### 4.2.10. Assessment of Biocompatibility

In order to test the biocompatibility of the collagen scaffolds, we used a human osteosarcoma cell line, MG63 (CRL-1427, ATCC, Manassas, VA, USA), frequently utilized to assess the cytological impact of biomaterials designed for bone repair. However, as the collagen sponges were not cross-linked, they rapidly dissolved upon immersion in the culture medium (low-glucose Dulbecco’s Modified Eagle Medium from Sigma Aldrich, St. Louis, MO, USA, supplemented with 10% (*v*/*v*) fetal bovine serum from Gibco BRL, Gaithersburg, MD, USA, and 100 IU/mL of penicillin, 100 µg/mL of streptomycin, 50 µg/mL neomycin, all from Sigma Aldrich, St. Louis, MO, USA). For this reason, biocompatibility was tested against the collagen gel, which was diluted 10 and 20 times in culture medium, with final concentrations of 1 mg/mL and 0.5 mg/mL, respectively. The final solutions were sterilized via filtration (0.2 μm pore size).

The impact of the collagen gels on MG63 cells was evaluated using an XTT assay (Thermo Fisher Scientific, Waltham, MA, USA), 72 h after adding the extracts. In short, cells were washed with PBS and incubated with 100 µL/well XTT working solution for 3 h at 37 °C, 5% CO_2_. After the incubation, the absorbance was read at 450 nm versus 690 nm using a TECAN spectrophotometer. The results were expressed as a percentage of the control (cells grown in culture medium without any sample addition). Data were analyzed using a One-way ANOVA and are presented as mean ± standard deviation (SD) (***, ### *p* < 0.001).

## Figures and Tables

**Table 1:** Proximate analysis of collagen gels extracted from different sources.

Sample *	Dry Substance Content **, %	Total Nitrogen **, %	Protein Content **^,^ ***, %	pH **, pH Units
COLL_C	1.90	0.32	1.80	2
COLL_B	1.65	0.27	1.52	4.2
COLL_T	1.60	0.27	1.52	3.0
COLL_P	1.15	0.18	1.13	3.0

* COLL_C—collagen extracted from calf hide, COLL_B—collagen extracted from American buffalo hide, COLL_T—collagen extracted from turkey skin, COLL_P—collagen extracted from perch skin. ** The proximate analysis of collagen gels was determined according to Romanian standard methods, described in Section 4. Materials and Methods, Section 4.2. Methods, Section 4.2.2. *** The nitrogen-to-protein conversion factor employed was 6.25 for perch gel and 5.62 for the other gels, in accordance with the standard methodology used.

**Table 2:** Thermogravimetric analysis of collagen, with data related to each decomposition stage.

Sample	Mass Loss, %			Residual Mass, %	T_max_ (DTG), °C
	25–100, °C	100–300, °C	300–500, °C		
Gel COLL_C	8.51	11.97	45.46	34.06	322
Gel COLL_B	8.43	16.38	48.64	26.55	315
Gel_COLL_T	6.95	15.23	49.72	28.10	323
Gel_COLL_P	5.80	28.86	33.78	31.56	309

**Table 3:** Determination of microbial contamination of collagen sponges from different sources.

Sample	TAMC * (CFU/g)	TYMC ** (CFU/g)	Detection of *E. coli*	Detection of *S. aureus*	Detection of *P. aeruginosa*
COLL_C	1.9 × 10^1^	1.2 × 10^1^	Absent	Absent	Absent
COLL_B	2.2 × 10^1^	1.2 × 10^1^	Absent	Absent	Absent
COLL_T	2.4 × 10^1^	1.5 × 10^1^	Absent	Absent	Absent
COLL_P	1.1 × 10^1^	1 × 10^1^	Absent	Absent	Absent

* TAMC is the total number of aerobic microorganisms: total aerobic microbial count. ** TYMC is the total number of yeasts and filamentous fungi: total combined yeasts and molds count.

## Data Availability

Data are contained within the article.
